# The impact of dynamic marketing capabilities (DMCs) and strategic information management (SIM) on international start-up success: A meta narrative analysis

**DOI:** 10.1016/j.heliyon.2024.e40541

**Published:** 2024-11-20

**Authors:** Cheryl Marlitta Stefia, Budhi Haryanto, Lilik Wahyudi, Ahmad Ikhwan Setiawan

**Affiliations:** a Universitas Sebelas Maret, Indonesia; b Universitas Kristen Duta Wacana, Indonesia

**Keywords:** Dynamic marketing capabilities, Strategic information management, International startups, Meta-narrative analysis, Global startup success

## Abstract

The success of international startups is profoundly influenced by their adeptness in utilizing dynamic marketing capabilities (DMCs) and strategic information management (SIM) to achieve competitive advantage and sustainable growth in global markets. This research examines the effects of these crucial elements on the success of international startups through a meta-narrative analysis. By synthesizing findings from 20 articles published in Scopus-indexed journals between 2020 and 2024, we aim to provide an in-depth understanding of the interaction between DMCs and SIM and their impact on startup performance in global markets. The research design includes an extensive review and synthesis of the literature on DMCs and SIM, emphasizing their importance in improving startup agility, market responsiveness, and strategic decision-making. Our findings suggest that startups are proficient in DMCs, such as adaptive market orientation and innovative marketing strategies, alongside robust information management systems, demonstrating higher success rates, accelerated growth, and a sustained competitive advantage in international markets. These insights highlight the pivotal role of integrating marketing dynamism with SIM for startup success. The global implications of this study suggest that international startups must prioritize the development of these capabilities to navigate the complexities of global markets effectively and ensure long-term viability. This research underscores the critical importance of strategic investments in marketing and information management to foster startup success globally.

## 1 Introduction

The rapid globalization and technological advancements have significantly transformed the business landscape, particularly for international startups. These firms must adeptly navigate dynamic environments and leverage strategic information management and marketing capabilities to ensure success [1]. The influence of globalization on entrepreneurship is profound, necessitating a deep understanding of diverse cultural and economic contexts [2]. Technological advancements driven by globalization foster innovation and reconfigure global supply chains, enabling startups to compete effectively on a global scale [3]. Moreover, Industry 4.0 technologies enhance the capabilities of startups, offering new opportunities for growth and market expansion [4]. For instance, the Indian startup ecosystem benefits from globalization through increased foreign investment and talent influx, driving innovation and economic growth [5]. Comprehending the relationship between globalization and technological progress is crucial for international startups to effectively maneuver through dynamic markets, foster innovation, and attain sustainable development.

The proficiency in adaptive marketing and tactical information management is crucial for worldwide startups to successfully manage the intricacies of a swiftly changing global landscape [6]. These proficiencies enable startups to adjust their marketing approaches in response to evolving consumer preferences and competitive dynamics, ensuring their pertinence in varied cultural and economic environments [7]. By effectively managing strategic information, startups can make informed decisions that align with technological advancements and globalization trends, fostering innovation and optimizing operations [8]. This synergy not only enhances their ability to compete on a global scale but also positions them to leverage emerging opportunities, ultimately driving sustainable growth and success in the international arena [9].

Meta-analyses have examined the effects of dynamic marketing capabilities (DMCs) and strategic information management (SIM) practices on startups, providing significant quantitative insights into their efficacy. DMCs, encompassing knowledge management skills, interface coordination abilities, and customer relationship management skills, have been shown to positively influence startup success [10]. Innovations in SIM also play a crucial role, functioning as an intervening variable between dynamic marketing abilities and organizational performance. Moreover, startups that effectively utilize DMCs and market orientation can establish long-term competitive advantages in rapidly changing environments [6]. Business accelerators have been demonstrated to improve the DMCs of startups, specifically in the areas of absorption, integration, and innovation. These enhancements substantially influence the performance of startups [11]. Moreover, effective knowledge management practices can help fintech startups develop DMCs, which in turn boosts resilience and performance [12,13]. These studies have offered substantial evidence regarding the critical role of DMCs and SIM innovations which encompass elements such as knowledge management skills, interface coordination abilities, customer relationship management skills, and effective knowledge management practices in improving startup performance, enhancing competitiveness, and ensuring sustainability.

However, there is a shortage of meta-narrative analyses in this field, leaving the narrative or experiential dimensions of DMCs and SIM implementation—and their impact on enhancing startup agility, market responsiveness, and strategic decision-making—largely unexplored. This gap limits our understanding of how these capabilities influence these key areas from an experiential perspective. This present research aims to bridge this gap by focusing on the narrative experiences of startups, thereby offering deeper insights into their strategic practices and decision-making processes. By doing so, it seeks to provide nuanced insights into how startups can integrate DMCs with SIM to achieve higher success rates, faster growth, and sustained competitive advantage in global markets. The implications of this study are significant, suggesting that international startups must prioritize the development of these capabilities to navigate the complexities of global markets effectively and ensure long-term viability.

## 2 Literature review

### 2.1 The role of dynamic marketing capabilities in shaping international startup success

Dynamic marketing capabilities (DMCs) are critical in helping international startups navigate the complexities of global markets. DMCs enable startups to respond effectively to the challenges presented by diverse and rapidly changing environments. By harnessing these capabilities, startups are better equipped to adjust to changing market conditions, foster innovation, and maintain competitive edges. The idea of DMCs is based on the dynamic capability framework, which highlights the company's ability to integrate, enhance, and reorganize internal and external skills to adapt to rapidly changing conditions. Prominent theories in this area include Teece's [14] framework on dynamic capabilities and Eisenhardt and Martin's [15] perspective on these capabilities in high-velocity markets. Additionally, strategic information management (SIM) plays a pivotal role, involving the collection, analysis, and application of data to inform strategic decision-making.

The significance of DMCs underscores their critical role for startups aiming to adapt and succeed in international markets. Research consistently highlights the favorable effects of DMCs on multiple performance indicators for startups. Evidence reveals that DMCs positively affect startup performance by improving market information management, fostering interface coordination, and enhancing client relationship management [10]. Furthermore, the capacity to interpret and react to market signals is crucial for startup growth, as it enables startups to swiftly adjust their strategies to meet changing customer demands, identify emerging market opportunities, and maintain a competitive edge [16].

Moreover, SIM amplifies the effectiveness of DMCs decision-making in optimizing startup operations. It acts as a mediating factor, providing feedback that further refines marketing strategies [10]. Effective information management is linked to improved decision-making processes and competitive advantages [17].

Globalization, as a broader context, exposes startups to a wider array of opportunities and challenges. In this landscape, the influence of international entrepreneurial culture (IEC) becomes particularly significant, as it plays a critical role in developing startups' dynamic capabilities and enhancing performance in turbulent markets [18]. By fostering adaptability and a proactive mindset, IEC helps startups leverage dynamic marketing capabilities to navigate international markets successfully.

Beyond cultural factors, technological capabilities are integral to fostering startup growth. While not the exclusive determinants, these capabilities are progressively essential for attaining scalability and advancing innovation. Technological capabilities enable firms to develop scalable and innovative business models [16]. Industry 4.0 technologies facilitate better market sensing and customer relationship management, critical components of DMCs [6].

Moreover, the economic and cultural contexts of emerging markets shape how startups develop and deploy DMCs. Understanding these contexts is essential for tailoring strategies that resonate with local markets. For instance, in Brazilian and Indian contexts, the ability to integrate entrepreneurial culture with dynamic capabilities is critical for achieving successful international performance, as it enables startups to adapt to diverse market conditions, innovate effectively, and compete in global markets [16,18].

Despite extensive research, there are still gaps in comprehending the intricate interaction between DMCs and other strategic factors across various cultural and economic settings. These gaps underscore the necessity for further investigation in the field. Additional studies are required to examine the role of technological integration and the influence of different market dynamics on the effectiveness of these capabilities. Case studies from Hebei, China, and Brazil illustrate how startups utilize DMCs and SIM to improve performance. These examples not only demonstrate the practical application of theoretical models but also offer valuable insights into effective international strategies [6,10].

In summary, the reviewed literature highlights the critical role of DMCs in determining the success of international startups. This synthesis reveals how various factors influencing startup performance are interrelated. When DMCs are combined with SIM and bolstered by an entrepreneurial culture, they significantly boost startup performance in international markets. Technological advancements and contextual factors also impact the effectiveness of these capabilities, emphasizing the necessity for flexible and innovative strategies. DMC are essential for international startups, enabling them to adapt to market changes, utilize strategic information, and sustain competitive advantages. This review underscores the interconnectedness of these capabilities with SIM and international entrepreneurial culture, offering a thorough understanding of their effects on startup performance.

### 2.2 Strategic information management: navigating global markets and enhancing competitiveness

Strategic Information Management (SIM) involves a systematic approach to collecting, analyzing, and utilizing information to support strategic decision-making and enhance competitive positioning in global markets [19]. Effective use of SIM allows organizations to navigate the complexities of the modern business environment, respond swiftly to market shifts, harness technological advancements, and sustain a competitive advantage through informed decisions based on accurate and timely data. The theoretical foundation of SIM is anchored in the Resource-Based View (RBV) and knowledge management frameworks. The RBV, primarily developed by Barney [20] and articulated in his seminal 1991 paper “Firm Resources and Sustained Competitive Advantage”, asserts that organizations can attain sustainable competitive advantages by efficiently managing and utilizing resources that are valuable, rare, unique, and irreplaceable.

Expanding upon this foundation, the RBV suggests that organizations can secure a long-term competitive edge by effectively managing and exploiting resources that are valuable, rare, and difficult to imitate [19]. In conjunction with this, knowledge management frameworks highlight the essential role of knowledge in enhancing organizational performance. These frameworks underscore the significance of generating, sharing, and applying knowledge within an organization to foster improved performance and innovation [21].

To implement SIM effectively, several key components must be considered, including data collection, analysis, dissemination, and feedback mechanisms. These components form the backbone of a robust information management system. Effective SIM involves ensuring that relevant information is gathered, processed, and shared across the organization to support strategic decision-making. For instance, timely and accurate data collection helps organizations identify market trends and customer needs, while data analysis provides insights that inform strategic initiatives [22]. Dissemination and feedback mechanisms ensure that information flows efficiently within the organization, facilitating better coordination and alignment of strategic goals [23].

Furthermore, effective SIM significantly enhances the performance of international startups by improving market responsiveness, customer engagement, and operational efficiency. The positive impacts of SIM are especially pronounced in the startup context, where agility is crucial. Startups that implement robust information management systems are better equipped to identify and capitalize on market opportunities, adapt to changes, and deliver superior customer value [24]. Additionally, the ability to manage information strategically helps startups streamline operations and reduce inefficiencies, leading to better overall performance [25].

Moreover, SIM and DMCs are closely intertwined, collectively enhancing a startup's ability to navigate dynamic environments. This relationship emphasizes the need for real-time adaptability in marketing strategies. DMCs involve the ability to adapt marketing strategies based on real-time information, which is facilitated by effective SIM [21]. This synergy allows startups to be more adaptable and attuned to market demands, improving their competitive positioning [23].

As globalization unfolds, it presents both challenges and opportunities for SIM. Navigating this landscape requires careful management of diverse information sources. On one hand, globalization increases the complexity of managing diverse information sources and adapting to various cultural contexts. On the other hand, it provides access to a broader range of data and insights, which can enhance strategic decision-making [26]. Effective SIM enables startups to leverage global data to better understand international markets and tailor their strategies accordingly [27].

In addition, advancing technologies such as big data, artificial intelligence (AI), and advanced analytics play a significant role in shaping SIM practices. These technologies are transforming the capabilities of startups in the information management arena. These technologies enable startups to process large volumes of data, generate predictive insights, and make more informed strategic decisions [22]. The integration of these technologies into SIM processes enhances a startup's ability to innovate and maintain a competitive edge in global markets [28].

Case studies from various industries illustrate the successful implementation of SIM practices. These real-world examples provide valuable insights into effective SIM strategies. For instance, a study on commercial banks in Turkey highlights how SIM, supported by effective information systems, improve operational performance and market responsiveness [23]. Another example from the tourism industry demonstrates how SIM helps organizations navigate competitive markets and achieve differentiation [29].

Despite its importance, startups face several challenges when implementing SIM strategies, including resource limitations, data integration issues, and cultural differences. Overcoming these challenges is essential for successful information management. Addressing these challenges requires a strategic approach to information management that leverages technology, fosters a culture of data-driven decision-making, and aligns with organizational goals [30]. Ultimately, SIM is essential for enhancing the competitiveness of international startups. By embracing SIM, startups position themselves for success in global markets. By effectively managing and leveraging information, startups can make data-driven decisions, respond to market fluctuations, and maintain a strategic advantage in global markets. The integration of emerging technologies and a strategic approach to information management are crucial for achieving sustained success.

## 3 Methodology

This research employs meta-narrative analysis to integrate findings from multiple narrative reviews on the effects of dynamic marketing capabilities and strategic information management on the success of international startups. The studies reviewed demonstrate that dynamic capabilities facilitate startups' adaptability by enabling them to tailor marketing strategies to different international contexts. This flexibility is essential for startups operating in competitive global markets, where responsiveness to cultural and economic variations significantly impacts success. The historical evolution of dynamic capabilities highlights the increasing importance of digital transformation for SMEs, as indicated by recent studies that link these capabilities to digital adaptation in globalized markets [31]. Findings from the strategic information management paradigm emphasize the role of information flow and risk management in supporting startups' growth and sustainability. By leveraging information-driven strategies, startups can navigate the complexities of international expansion, minimizing risks and adapting swiftly to regulatory changes. Research highlights the significant role that strategic information management plays in enterprise risk assessment and international expansion planning [32]. Additionally, studies reveal that economic and institutional factors shape entrepreneurial decision-making, suggesting that startups must tailor their approaches to the specific regulatory and market conditions of each country [33]. These findings underscore the interconnectedness of dynamic marketing capabilities and strategic information management as critical elements for international startup success. Both paradigms illustrate how adaptability and informed decision-making contribute to sustainable growth in diverse international contexts.

The meta-narrative approach is well-suited for this study because it allows for the amalgamation of insights from various narrative reviews, offering a thorough understanding of intricate research issues. The study adheres to the ‘meta-narrative’ procedures established by Greenhalgh et al. [34], with each phase significantly contributing to the understanding of dynamic marketing capabilities and strategic information management. In the planning phase, we established research questions centered on how dynamic marketing capabilities and strategic information management influence the success of international startups. This phase also included defining initial inclusion criteria to guide the literature search. Moving to the search phase, a comprehensive literature search was conducted across key databases, including Scopus, with a focus on publications from 2020 to 2024.

**Fig. 1:**
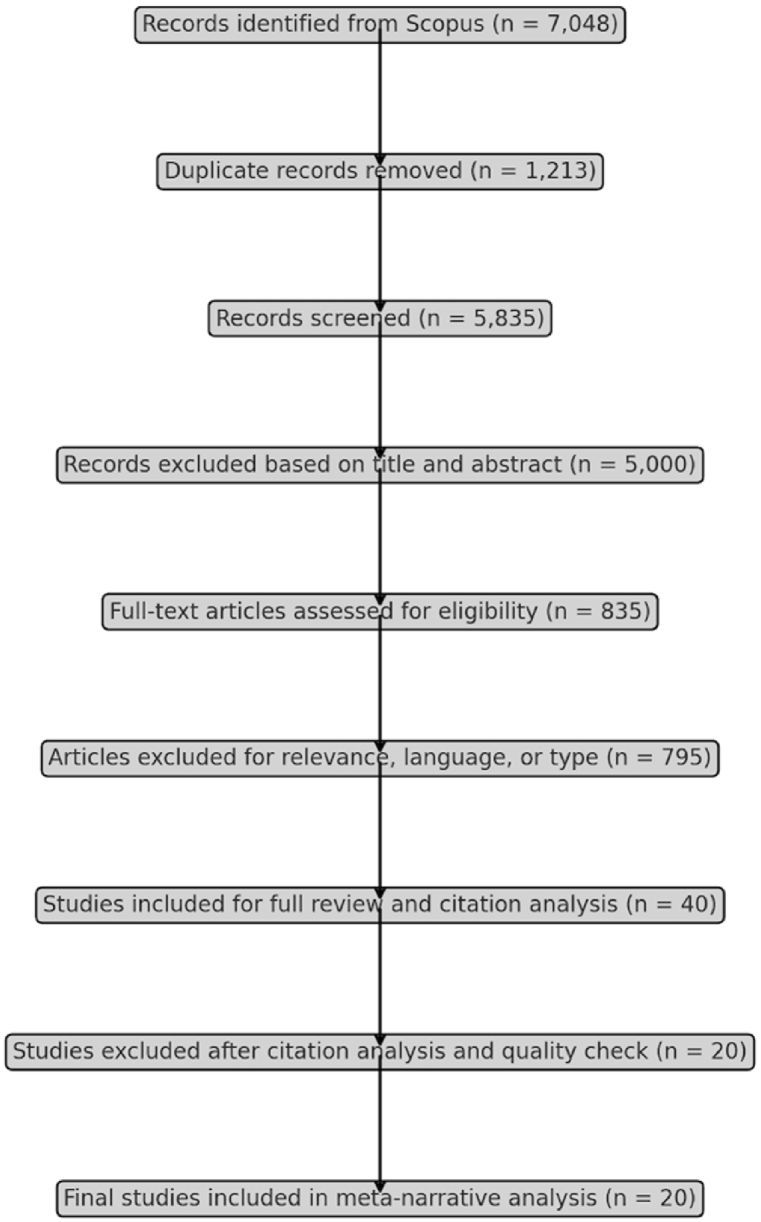
PRISMA flowchart of article selection process.

To ensure a rigorous and transparent selection of articles, this study utilized the PRISMA (Preferred Reporting Items for Systematic Reviews and Meta-Analyses) framework as a quality assessment tool. PRISMA's structured guidelines facilitated a systematic approach to identifying, screening, and selecting studies relevant to the research objectives. Initially, an electronic search through the Scopus database yielded 7,048 articles related to dynamic marketing capabilities and strategic information management in the context of international startups. Titles and abstracts were reviewed to filter out duplicates and exclude studies not directly aligned with the research focus. The eligibility criteria were then applied, which included relevance to the core research questions, publication in English, and exclusion of conference proceedings, review articles, and reports. This stringent filtering ensured that only the most credible empirical studies were retained. Following this, each selected article underwent a full-text review to confirm its alignment with the study's objectives. A citation analysis was also performed to verify the scholarly impact and domain relevance of each study, ultimately leading to a refined selection of 20 high-quality articles for in-depth synthesis. A PRISMA flowchart summarizing the selection process is provided in Fig. 1.

In the mapping phase, we identified the key elements of each research paradigm to elucidate the contributions of dynamic marketing capabilities and strategic information management. For dynamic capabilities, the key conceptual framework emphasizes flexibility and market adaptation in global contexts, with theoretical elements encompassing strategic agility and responsiveness. Methodologically, this includes case studies and empirical research on marketing adaptations across various markets. In contrast, the focus of strategic information management is on data-driven approaches to risk assessment and decision-making. This mapping phase also revealed dominant terminologies across these traditions, such as “market agility” and “information-driven strategies,” which highlight how these concepts shape our understanding of international startup success.

During the appraisal phase, each selected study underwent evaluation for validity and relevance. This involved a careful scrutiny of each article for its empirical contributions to the understanding of dynamic capabilities and information management. Finally, in the synthesis phase, the findings from the selected articles were synthesized to construct a comprehensive narrative regarding the roles of dynamic marketing capabilities and strategic information management in the success of international startups. This synthesis took into account the historical and theoretical contexts of each tradition, as well as empirical findings that underscore the importance of adaptability and information flow in achieving competitive advantage. Table 1 outlines the meta-narrative analysis process adapted for this study.

**Table 1:** Phases in meta-narrative analysis, according to Greenhalgh et al. [34].

Phases in meta-narrative analysis
(1) Planning phase
1. (a) Outline the initial research questions.
(2) Search phase
1. (a) Electronically search for papers in key databases,
2. (b) Search for seminal papers by tracking citations of references. Evaluate these according to comprehensiveness and contribution to work within the tradition
(3) Mapping phase
Identify:
1. (a) The key elements of the research paradigm (conceptual, theoretical, methodological),
2. (b) The main findings of the research,
3. (c) The prevailing language used to describe and define the concept.
(4) Appraisal phase
1. (a) Evaluate each study for its validity and relevance to the research questions,
2. (b) Extract and collate the key results.
(5) Synthesis phase
1. (a) Identify the key dimensions of the concept that has been researched,
2. (b) Analyze the conceptual and descriptive concepts through different types of definition,
3. (c) Consider the dimensions in turn and give a narrative account of the contributions.

**Table 2:** The key details and findings from the literature on dynamic marketing strategies and information management practices to the agility and performance of startups.

Author(s)	Main Theme	Key Findings
[18]	Dynamic Marketing Capabilities, International Performance, Entrepreneurial Culture, Innovation	Dynamic capabilities facilitate international performance through entrepreneurial culture and ambidextrous innovation
[35]	Dynamic Capabilities, International Expansion	Dynamic capabilities mediate international expansion and performance
[36]	Dynamic Capabilities, Innovation, Branding Capabilities, Competitive Advantage	Strategic information management coupled with innovation and branding capabilities drives compe
[37]	Marketing Capabilities, International Performance, Technological Turbulence	Marketing capabilities improve performance via marketing communication and are influenced by technological turbulence.
[38]	Marketing Agility, Emerging Markets, Advanced Markets	Marketing agility is essential for the performance of firms from emerging markets in advanced markets.
[39]	Digital Marketing Capabilities, International Firms	Digital marketing capabilities create relational advantages in international markets
[40]	Internet Marketing Capabilities, International Market Performance	Internet marketing capabilities significantly influence international market performance
[41]	Dynamic Capabilities, International Strategies, Emerging Markets	Dynamic capabilities support the international strategies of emerging market multinationals
[16]	Dynamic Marketing Capabilities, Startup Growth	Dynamic capabilities significantly contribute to startup growth trajectories
[42]	Dynamic Marketing Capabilities, Adaptive Capabilities, SME Performance	Dynamic and adaptive marketing capabilities enhance SME performance in international markets
[43]	Dynamic Capabilities, Knowledge Management	Knowledge management and behavioral supports building dynamic marketing capabilities
[44]	Dynamic Capabilities, Knowledge Management	Knowledge management supports building dynamic marketing capabilities
[45]	Dynamic Capabilities, International Performance, Entrepreneurial Culture	Entrepreneurial culture enhances the effectiveness of dynamic capabilities in emerging market ventures
[8]	Dynamic Marketing Capabilities, Marketing Leadership, International Performance	Dynamic marketing capabilities improve international performance moderated by marketing leadership
[46]	Dynamic Marketing Capabilities, E-commerce, Emerging Markets	Digital marketing capabilities are essential for international market performance in e-commerce
[47]	Intellectual Capital, Knowledge Management, International Trade	Knowledge management and business processes mediate the impact of intellectual capital on international trade
[10]	Dynamic Marketing Capabilities, Strategic Information Management, International Performance	Strong dynamic marketing capabilities and strategic information management practices enhance international startup per
[6]	Dynamic Capabilities, Market Orientation, Dynamic Environments	Effective information management supports the development of adaptive marketing strategies
[48]	Dynamic Marketing Capabilities, Pandemic Performance, Export Manufacturers	Dynamic marketing capabilities helped Polish manufacturers maintain performance during the pandemic
[49]	Dynamic Marketing Capabilities, Legitimacy, International Markets	Dynamic marketing capabilities are critical for establishing legitimacy in international markets

This study conducted a comprehensive meta-narrative analysis of articles listed in Table 3, arranged chronologically from 2020 to 2023. The analysis aimed to synthesize insights on the influence of dynamic marketing capabilities and strategic information management on the performance of international startups.

**Table 3:** The key details and findings from the literature on dynamic marketing strategies and information management practices to market responsiveness and performance of startups.

Author(s)	Main Themes	Key Findings
[18]	International Entrepreneurial Culture, DMC	International entrepreneurial culture enhances DMC, improving international venture performance.
[35]	Dynamic Capabilities, International Expansion	DMC mediates the relationship between strategic management and international expansion.
[36]	Innovation, Branding Capabilities, Competitive Advantage	Innovation and branding capabilities, moderated by entrepreneurial orientation, improve SME performance.
[37]	Marketing Capabilities, Technological Turbulence	DMC and marketing communication mediate the relationship between marketing capabilities and performance.
[38]	Marketing Agility, Emerging Markets	Marketing agility is crucial for firms from emerging markets in advanced markets.
[39]	Digital Marketing Capabilities, International Firms	Digital marketing capabilities improve international firms' relational performance.
[40]	Internet Marketing Capabilities, International Performance	Internet marketing capabilities drive international market performance.
[41]	Dynamic Capabilities, Emerging Market Multinationals	Dynamic capabilities support international strategies of emerging market multinationals.
[16]	Internet Marketing Capabilities, International Performance	Internet marketing capabilities drive international market performance.
[42]	Dynamic Capabilities, Startup Growth	Dynamic capabilities facilitate startup growth by enhancing strategic flexibility.
[43]	Dynamic and Adaptive Marketing Capabilities, Performance	Adaptive marketing capabilities are crucial for performance in B2B international markets.
[44]	Dynamic Capabilities, Knowledge Management	Building dynamic capabilities supports international marketing knowledge management.
[45]	Dynamic Capabilities, Entrepreneurial Culture	Entrepreneurial culture impacts the effectiveness of dynamic capabilities in emerging markets.
[8]	Dynamic Marketing Capability, Marketing Leadership	DMC enhances marketing performance, moderated by marketing leadership.
[46]	Dynamic Marketing Capabilities, E-commerce	DMC of small businesses enhance performance on e-commerce platforms.
[47]	Intellectual Capital, Knowledge Management	Intellectual capital and knowledge management impact international trade.
[10]	Dynamic Marketing Capabilities (DMC), Strategic Information Management (SIM), International Startup Performance	DMC and SIM drive international startup performance through improved market responsiveness.
[6]	Dynamic Capabilities, Market Orientation	Effective dynamic capabilities and market orientation improve startup performance in dynamic environments.
[48]	Dynamic Marketing Capabilities, Pandemic Impact	DMC influences international performance during the pandemic.
[49]	Dynamic Marketing Capabilities, Legitimacy	DMC boosts legitimacy in international markets.

## 4 Findings and discussion

The meta-narrative analysis uncovered several crucial insights into how DMCs and SIM affect the success of international startups.

### 4.1 Start-up agility

The synthesis of literature on DMCs and SIM reveals a nuanced yet cohesive framework for understanding startup agility and performance, particularly in international contexts. This synthesis moves beyond a simple summary of individual studies to develop higher-order concepts that highlight the interconnected roles of dynamic marketing strategies and information management practices in fostering agility and adaptability among startups. The interdependence between DMCs and SIM emerges as a critical factor, enhancing the ability of startups to adapt swiftly to evolving market conditions and seize emerging opportunities. The data relevant to this analysis is presented in Table 2.

According to Table 2, the principal themes identified from the narrative analysis include DMC, SIM, international performance, innovation and entrepreneurial culture, and digital and internet marketing. These themes collectively underscore the key factors that drive the success of international startups in a rapidly evolving business environment.

Studies consistently point to the role of these capabilities in driving international expansion, facilitated by elements like entrepreneurial culture, ambidextrous innovation, and a strong digital marketing foundation. For example, Guo and Hartini [10] highlight DMC, emphasizing the interconnected relationship between these capabilities and SIM and their combined effect on the performance of international startups. Teixeira et al. [16] and Buccieri et al. [45] examine how dynamic capabilities support startup growth and enhance international performance by allowing firms to quickly adapt to market opportunities and threats. They underscore that startups with robust dynamic capabilities, integrated with strategic information management, often outperform their peers in international markets, demonstrating more substantial growth trajectories and enhanced market orientation.

The literature also highlights the mediating effects of innovation, entrepreneurial culture, and digital marketing tools, emphasizing their importance in amplifying the effectiveness of dynamic capabilities. The combination of dynamic marketing strategies with information management enables startups not only to establish competitive advantages but also to navigate technological turbulence and shifting consumer demands. For instance, the works of Ribeiro et al. [6] and Scuotto et al. [44] illustrate how strategic information management strengthens market orientation in dynamic environments, suggesting that the capacity to leverage information effectively is foundational to achieving agility. Ferreira and Coelho [38] find that the integration of strategic information management with innovation and branding capabilities significantly enhances a startup's ability to gain a competitive advantage.

Ledesma-Chaves et al. [35] and Reimann et al. [42] explore international performance, emphasizing the intermediary role of dynamic capabilities in facilitating international expansion and enhancing performance. They show that dynamic capabilities enhance international performance, mediated by strategic elements like innovation and branding. Additionally, Chatterjee et al. [8] and Martin et al. [37] underscore the importance of dynamic marketing capabilities in advancing worldwide marketing success, noting that their effectiveness is influenced by technological turbulence.

Innovation and entrepreneurial culture are vital for leveraging dynamic capabilities to improve performance. Buccieri et al. [45] and Deng et al. [41] highlight the significance of an entrepreneurial culture and ambidextrous innovation in this context. Digital and internet marketing are increasingly important in the global marketplace. Kim and Lim [46] and Liu et al. [40] examine the influence of internet marketing capabilities on worldwide marketing success, emphasizing the growing importance of digital tools in marketing strategies. Furthermore, in the face of rapidly digitalizing markets, the studies by Wang [39] and Rivero-Gutiérrez et al. [49] reveal that strong digital marketing capabilities help establish legitimacy and foster relational advantages in international markets, thereby supporting global market entry and reputation building. They indicate that digital marketing proficiency is not only crucial for gaining relational advantages but also essential for establishing legitimacy in global markets, supporting reputation-building efforts and fostering sustainable growth.

The synthesis of these narratives presents a nuanced yet cohesive understanding of how dynamic marketing strategies and information management practices affect startup agility. The interconnection between dynamic marketing capabilities and strategic information management is evident, as effective information management enhances the development of adaptive marketing strategies. However, contradictions and challenges emerge, with some studies focusing on the direct impact of dynamic capabilities on performance, while others emphasize the mediating and moderating roles of factors such as innovation culture and technological turbulence. The analysis reveals that the connection between dynamic capabilities and performance is frequently mediated by factors such as innovation, entrepreneurial culture, and digital marketing proficiency. These findings suggest that a comprehensive approach is essential for startups to achieve agility and maintain sustained performance.

A case study example provided by Guo and Hartini [10] offers an empirical analysis showing how startups with strong dynamic marketing capabilities and strategic information management practices outperform their peers in international markets. Supporting evidence from Teixeira et al. [16] presents data on Brazilian startups, demonstrating that dynamic capabilities significantly contribute to their growth trajectories. Empirical insights from Ribeiro et al. [6] provide further understanding of how startups operationalize dynamic capabilities to adapt to rapidly changing market conditions, enhancing their market orientation and performance.

Similar perspectives are seen in the works of Buccieri et al. [45] and Ledesma-Chaves et al. [35], both of which agree on the importance of dynamic capabilities in international expansion, although they highlight different mediating factors such as entrepreneurial culture and innovation. Differing views are noted in the studies by Kim and Lim [46] and Deng et al. [41], with the former focusing on the digital aspect of marketing capabilities and the latter emphasizing traditional dynamic capabilities and their role in international strategy.

For practitioners, the findings suggest that investing in dynamic marketing capabilities and robust information management systems is crucial for enhancing startup agility and international performance. Additionally, fostering an entrepreneurial culture and embracing digital marketing tools are key strategies for success. The analysis enriches theoretical understanding by integrating diverse perspectives on dynamic capabilities, strategic information management, and their combined impact on startup agility and performance. It underscores the need for a multifaceted approach to studying these phenomena. The meta-narrative analysis confirms that dynamic marketing capabilities and strategic information management are pivotal in improving startup agility and performance, especially in international contexts. The findings challenge prior understandings by highlighting the mediating role of innovation, entrepreneurial culture, and digital marketing capabilities. Future research should explore these relationships further to provide deeper insights into the mechanisms driving startup success in dynamic and competitive environments.

Contradictions across studies reflect the complexity of these interrelationships, with some research focusing on the direct effects of dynamic capabilities on performance, while others emphasize mediating factors such as innovation culture and technological adaptation. This complexity underscores the need for a holistic, multifaceted approach to studying dynamic capabilities in startups, as different aspects of capability and information management are emphasized across varying market contexts. The integration of these perspectives enriches theoretical understanding and offers practical insights, suggesting that for startups to achieve sustained agility and performance, they must not only develop dynamic marketing capabilities but also invest in comprehensive information management systems and foster a culture of innovation. By examining these capabilities in combination, this synthesis presents a framework for practitioners to understand how to leverage both marketing agility and strategic information for competitive advantage in volatile and international markets. Future research should further investigate these relationships, particularly how innovation, digital marketing, and entrepreneurial culture mediate the connection between dynamic capabilities and startup success.

This study emphasizes the crucial importance of integrating dynamic marketing capabilities with strategic information management for the success of international startups. The synergistic relationship between these capabilities emphasizes the necessity for a holistic approach to capability development [10]. Dynamic marketing capabilities empower startups to adapt to market changes and capitalize on emerging opportunities. These capabilities are crucial for maintaining competitiveness in dynamic and uncertain environments [41]. However, their effectiveness is significantly enhanced when complemented by robust strategic information management systems [43].

### 4.2 Market responsiveness

The meta-narrative analysis of literature on dynamic marketing strategies and strategic information management (SIM) presents a comprehensive framework that elucidates how these elements enhance strategic decision-making and performance, especially within startups and international ventures. Studies examined within this analysis emphasize the interconnectedness of dynamic marketing capabilities (DMC) and SIM, exploring their combined impact on startups' market responsiveness, flexibility, and ability to capitalize on international growth opportunities. As detailed in Table 3, key findings reveal that DMC, often augmented by SIM, empowers firms to adapt to volatile markets through constant intelligence gathering, innovative marketing agility, and the rapid pivoting of strategies based on evolving trends and customer needs.

According to Table 3, the literature analysis identifies several key themes: Dynamic Marketing Capabilities (DMC), Strategic Information Management (SIM), Strategic Decision-Making, and Performance Outcomes. The objective of this meta-narrative analysis is to thoroughly review and synthesize literature on dynamic marketing strategies and information management practices and their effects on improving strategic decision-making. This analysis covers various studies examining the interplay between DMC, SIM, and performance outcomes, particularly within startups and international ventures. The findings are significant as they offer a detailed understanding of how these strategies can enhance strategic decision-making, providing valuable insights for both researchers and practitioners.

The analysis of the literature reveals several key themes: Dynamic Marketing Capabilities (DMC), Strategic Information Management (SIM), Strategic Decision-Making, and Performance Outcomes. The narrative around DMC emphasizes its critical role in enabling firms to adapt to volatile market environments. Studies by Guo and Hartini [10] and Buccieri et al. [18] illustrate how DMC facilitates innovation and strategic flexibility by enabling firms to continuously gather and analyze market intelligence, adapt their offerings based on customer feedback, and swiftly pivot their strategies in response to emerging trends and competitive pressures. This proactive approach allows firms to stay ahead of market dynamics and capitalize on new opportunities effectively. These capabilities are essential for maintaining competitive advantage and achieving sustained growth.

The narrative on SIM underscores its importance in enhancing decision-making processes, wherein effective SIM involves the systematic collection, analysis, and dissemination of information. This process strengthens strategic initiatives and operational efficiency, aligning marketing efforts with broader organizational goals, as emphasized by Teixeira et al. [16] and Ribeiro et al. [6]. Such alignment is essential for enhancing strategic decision-making, positioning firms to respond effectively to market shifts.

Strategic decision-making is depicted as a crucial outcome of the interplay between DMC and SIM. Studies such as those by Ledesma-Chaves et al. [35] and Ferreira and Coelho [38] provide evidence that firms with robust DMC and SIM frameworks are better positioned to anticipate and react to market shifts, leading to enhanced performance and informed decision-making.

The narrative here focuses on how startups, particularly those operating internationally, leverage DMC and SIM to overcome challenges and capitalize on opportunities. Research by Chatterjee et al. [8] and Reimann et al. [42] demonstrates that these capabilities are crucial for enabling startups to achieve growth and scalability in global markets, as they provide the flexibility and responsiveness needed to adapt to rapidly changing international demands.

Synthesizing these narratives reveals that DMC and SIM are interdependent and collectively enhance strategic decision-making. While Dynamic Marketing Capabilities (DMC) provide the agility needed to adapt marketing strategies, Strategic Information Management (SIM) ensures that these strategies are informed by precise and timely information. This synergy is crucial for firms operating in dynamic environments, as it enables them to remain competitive and make effective strategic decisions. Guo and Hartini [10] provide a detailed examination of the coupling relationship between DMC and SIM, highlighting how these capabilities drive international startup performance. Their study offers a concrete example of a startup leveraging these capabilities to navigate market uncertainties and achieve growth. Similarly, the research by Buccieri et al. [18] illustrates the role of international entrepreneurial culture in enhancing the effectiveness of DMC, thereby improving venture performance. This study underscores the importance of fostering a culture that supports innovation and adaptability.

Comparing findings across studies reveals both commonalities and divergences in perspectives. For instance, while Guo and Hartini [10] demonstrate that strategic information management directly improves startup performance through streamlined processes and enhanced decision-making. Meanwhile, Teixeira et al. [16] underscore the broader impact of dynamic capabilities on organizational growth, showing how these capabilities facilitate scalability and adaptability in evolving markets.

For practitioners, the findings suggest that investing in DMC and SIM is essential for achieving effective strategic decision-making and competitive advantage. Firms should prioritize the development of adaptable marketing strategies and robust information management systems to improve their capacity to respond effectively to market changes. Theoretically, this analysis enriches the understanding of the interplay between dynamic capabilities and information management in strategic decision-making. It highlights the need for further research into how these capabilities can be integrated to drive performance, particularly in the context of startups and international ventures.

The meta-narrative analysis supports the view that dynamic marketing capabilities and strategic information management are critical for improving strategic decision-making. These findings challenge conventional views of static marketing strategies and highlight the significance of agility and informed decision-making. Future research should examine the long-term effects of these capabilities on performance and explore optimal methods for firms to develop and integrate these practices.

This analysis also extends theoretical perspectives on the interdependence between dynamic capabilities and information management, enriching scholarly understanding of how these capabilities collectively drive strategic decision-making. Future research could explore the long-term effects of DMC and SIM on organizational performance, particularly in the startup and international contexts, to further clarify optimal methods for developing and integrating these capabilities. Ultimately, this meta-narrative analysis reinforces that integrating DMC with SIM is critical for international startup success, as SIM not only provides the market intelligence needed for strategic decision-making but also facilitates proactive market adaptation, enhancing the effectiveness of dynamic marketing strategiesally, the mediating role of SIM in linking DMC to performance outcomes underscores its importance as a foundational capability for startups navigating complex and volatile global markets. Strategic information management equips startups with the vital market intelligence needed for informed decision-making. It facilitates proactive market sensing and responsive actions, thereby augmenting the effectiveness of dynamic marketing strategies [50]. Furthermore, the mediating role of information management in the relationship between dynamic marketing capabilities and performance further highlights its significance [16].

### 4.3 Strategic decision-making

The meta-narrative analysis of literature on strategic decision-making reveals that DMC and SIM play an interdependent role in enhancing decision-making processes within organizations, particularly in startups and international ventures. DMC, encompassing the ability to adapt and restructure marketing strategies in response to changing market conditions, is essential for firms aiming to remain agile and responsive in dynamic environments. The data can be seen in Table 4.

**Table 4:** The key details and findings from the literature on dynamic marketing strategies and information management practices to strategic decision-making and performance of startups.

Author(s)	Main Themes	Key Findings
[18]	International Entrepreneurial Culture, DMC	International entrepreneurial culture enhances DMC, improving strategic decision-making and venture performance.
[35]	Dynamic Capabilities, International Expansion	DMC mediates the relationship between strategic management and international expansion, enhancing decision-making.
[36]	Innovation, Branding Capabilities, Competitive Advantage	Innovation and branding capabilities, moderated by entrepreneurial orientation, improve strategic decision-making and SME performance.
[37]	Marketing Capabilities, Technological Turbulence	DMC and marketing communication mediate the relationship between marketing capabilities and strategic decision-making.
[38]	Marketing Agility, Emerging Markets	Marketing agility is crucial for strategic decision-making in emerging market firms in advanced markets.
[39]	Digital Marketing Capabilities, International Firms	Digital marketing capabilities improve strategic decision-making and relational performance of international firms.
[40]	Internet Marketing Capabilities, International Performance	Internet marketing capabilities drive strategic decision-making and international market performance.
[41]	Dynamic Capabilities, Emerging Market Multinationals	Dynamic capabilities support strategic decision-making in international strategies of emerging market multinationals.
[16]	Internet Marketing Capabilities, International Performance	Internet marketing capabilities drive strategic decision-making and international market performance.
[42]	Dynamic Capabilities, Startup Growth	Dynamic capabilities facilitate startup growth and improve strategic flexibility.
[43]	Dynamic and Adaptive Marketing Capabilities, Performance	Adaptive marketing capabilities are crucial for strategic decision-making and performance in B2B international markets.
[44]	Dynamic Capabilities, Knowledge Management	Building dynamic capabilities supports strategic decision-making in international marketing knowledge management.
[45]	Dynamic Capabilities, Entrepreneurial Culture	Entrepreneurial culture impacts the effectiveness of dynamic capabilities in strategic decision-making in emerging markets.
[8]	Dynamic Marketing Capability, Marketing Leadership	DMC enhances strategic decision-making and marketing performance, moderated by marketing leadership.
[46]	Dynamic Marketing Capabilities, E-commerce	DMC of small businesses enhance strategic decision-making and performance on e-commerce platforms.
[47]	Intellectual Capital, Knowledge Management	Intellectual capital and knowledge management impact strategic decision-making in international trade.
[10]	DMC, SIM, Strategic Decision-Making	Coupling DMC and SIM enhances international startup performance and strategic decision-making.
[6]	Dynamic Capabilities, Market Orientation	Effective dynamic capabilities and market orientation improve strategic decision-making in dynamic environments.
[48]	Dynamic Marketing Capabilities, Pandemic Impact	DMC influences strategic decision-making and performance during the pandemic.
[49]	Dynamic Marketing Capabilities, Legitimacy	DMC boosts legitimacy in international markets through improved strategic decision-making.

Based on Table 4, the analysis of the literature reveals several key themes: Dynamic Marketing Capabilities (DMC), Strategic Information Management (SIM), Strategic Decision-Making, and Performance Outcomes. DMC encompass the ability to adapt and reconfigure marketing strategies in response to market changes, whereas SIM involves the effective management of information to support strategic decision-making. Strategic decision-making refers to the process of making informed decisions that shape the strategic direction of organizations, while performance outcomes pertain to the effects of DMC and SIM on organizational performance, particularly within international and startup contexts.

The narrative on DMC highlights its essential role in enabling firms to adapt to rapidly changing market conditions. Studies by Guo and Hartini [10] and Buccieri et al. [18] demonstrate how DMC provide firms with strategic flexibility and innovation, which are crucial for effective decision-making. These capabilities enable firms to rapidly adapt their strategies in response to emerging information and evolving market trends.

SIM is portrayed as a critical component in supporting strategic decision-making. Research by Teixeira et al. [16] and Ribeiro et al. [6] emphasizes that SIM involves the systematic collection, analysis, and dissemination of information, which is vital for making informed strategic decisions. Effective SIM practices ensure that decision-makers have access to relevant, accurate, and timely information.

The interconnection between DMC and SIM consistently emerges across studies, highlighting that organizations with robust frameworks integrating both capabilities are better positioned to make strategic decisions that enhance performance and competitive advantage. For instance, Ledesma-Chaves et al. [35] and Ferreira and Coelho [38] that firms leveraging both DMC and SIM frameworks can respond proactively to market shifts and capitalize on new opportunities. This dynamic interplay underscores a recurring theme in the literature: while DMC grants the agility required to pivot strategies, SIM provides the foundational information necessary for well-informed decision-making. The synergy between these capabilities is especially valuable in fast-paced markets, where strategic flexibility and data-driven insights are paramount to sustained success.

The influence of DMCs and SIM on performance outcomes is a recurring theme. Research by Reimann et al. [42] and Chatterjee et al. [8] demonstrates that these capabilities contribute to improved performance in both domestic and international markets. Improved decision-making processes directly contribute to enhanced organizational performance.

Synthesizing these narratives reveals that DMC and SIM are interdependent and collectively enhance strategic decision-making. While DMC provides the agility and flexibility needed to adapt strategies, SIM ensures that these strategies are based on sound and timely information. This synergy is crucial for organizations operating in dynamic environments, enabling them to make strategic decisions that drive performance and competitive advantage.

Guo and Hartini [10] provide a comprehensive examination of how the coupling of DMC and SIM enhances international startup performance by improving strategic decision-making. Their study offers a concrete example of a startup using these capabilities to navigate market uncertainties effectively. Similarly, the research by Buccieri et al. [18] highlights the role of international entrepreneurial culture in amplifying the effectiveness of DMC, leading to superior decision-making and performance outcomes. This study underscores the importance of an entrepreneurial culture in leveraging dynamic capabilities for strategic advantage.

Comparing findings across studies reveals both commonalities and divergences in perspectives. While Guo and Hartini [10] focus on the synergy between DMC and SIM in international startups, Teixeira et al. [16] emphasize the broader implications of dynamic capabilities on organizational growth and strategic decision-making. These differing narratives provide a more comprehensive understanding of how DMC and SIM impact strategic decisions.

For practitioners, the findings suggest that investing in DMC and SIM is crucial for enhancing strategic decision-making. Organizations should focus on developing these capabilities to improve their ability to make informed strategic decisions, ultimately leading to better performance and competitive advantage. Theoretically, this analysis enriches the understanding of the interplay between dynamic capabilities and information management in strategic decision-making. It highlights the need for further research into how these capabilities can be integrated to drive strategic decisions and performance, particularly in dynamic and competitive environments. The meta-narrative analysis supports the view that dynamic marketing capabilities and strategic information management are critical for improving strategic decision-making. These findings challenge traditional notions of static marketing strategies and underscore the importance of agility and informed decision-making in achieving performance outcomes. Future research should explore the long-term impacts of these capabilities on strategic decision-making and investigate how organizations can best develop and integrate these practices.

The contextual factors identified in this study indicate that integrating these capabilities is especially crucial in highly competitive and dynamic markets. Startups in such environments must focus on developing both dynamic marketing capabilities and strategic information management to secure a sustained competitive advantage [42]. Overall, the study's findings offer valuable insights for startup managers and policymakers, emphasizing the need to invest in both dynamic marketing capabilities and strategic information management to boost startup performance in global markets.

## 5 Conclusion

This study underscores the pivotal role of integrating dynamic marketing capabilities with strategic information management for the success of international startups. The meta-narrative analysis reveals that these capabilities interact synergistically, enhancing startups' agility, market responsiveness, and overall performance. The findings suggest that international startups must prioritize the development of these capabilities to navigate the complexities of global markets effectively and ensure long-term viability. The implications of this study are substantial for both researchers and practitioners. For researchers, it offers a thorough understanding of the interaction between dynamic marketing capabilities and strategic information management, identifying areas for further investigation. For practitioners, the findings offer actionable insights into how startups can enhance their performance by integrating these capabilities. Policymakers can also benefit from the study by developing supportive frameworks that encourage capability development in startups, thereby fostering innovation.

While DMCs and SIM play pivotal roles in shaping international startup success, the existing literature is limited by several methodological and contextual factors. Many studies rely heavily on quantitative metrics, often omitting critical narrative and experiential insights, which could provide a more nuanced understanding of how startups implement and benefit from these capabilities in practice. Furthermore, there is a pronounced publication bias toward studies showcasing positive impacts, while studies reporting neutral or negative findings are underrepresented. Future research should incorporate mixed methods, combining quantitative and qualitative analyses, to capture the full spectrum of DMCs and SIM implementation experiences across different cultural and economic contexts. Additionally, studies should aim to isolate the effects of DMCs and SIM independently, enabling a clearer understanding of each capability's unique contributions to startup performance. Finally, exploring alternative models that include other moderating factors, such as organizational culture, market stability, and resource availability, could provide a more comprehensive picture of how these capabilities contribute to or hinder international startup success.

## CRediT authorship contribution statement

**Cheryl Marlitta Stefia:** Writing – review & editing, Writing – original draft, Visualization, Resources, Project administration, Methodology, Formal analysis, Data curation, Conceptualization. **Budhi Haryanto:** Writing – review & editing, Writing – original draft, Visualization, Resources, Methodology, Formal analysis, Conceptualization. **Lilik Wahyudi:** Writing – review & editing, Writing – original draft, Supervision, Resources, Methodology, Formal analysis, Conceptualization. **Ahmad Ikhwan Setiawan:** Writing – review & editing, Writing – original draft, Supervision, Resources, Methodology, Formal analysis, Conceptualization.

## Data availability statement

Data included in article/supplementary material is referenced in the article.

## Declaration of competing interest

The authors declare that they have no known competing financial interests or personal relationships that could have appeared to influence the work reported in this paper.

## References

[1] Akaiso D., Markova M. (2023).

[2] Kyove J., Streltsova K., Odibo U., Cirella G. (2021).

[3] Milenković D., Petković J., Marinković S. (2022). 41st International Conference on Organizational Science Development.

[4] Thanh T.L., Mohiuddin M., Quang H.N. (2022). Impact of uncertainty and start-up opportunities on technopreneurial start-up success in emerging countries. Transnatl. Corp. Rev..

[5] Han H., Kim J., Kim D., Lee S.H., Pek J. (2021).

[6] Ribeiro A.M.S., Lacerda R.T. de O., Becker M. (2023). Operationalizing dynamic capabilities and market orientation: empirical insights for startups in dynamic environments. Int. J. Bus. Manag..

[7] Acevedo-Gelves L.K., Albornoz-Arias N. (2020). Theoretical review of dynamic capabilities. Pensamiento Gestión.

[8] Chatterjee S., Chaudhuri R., Vrontis D. (2022). Examining the marketing performance of the firms from an international dynamic marketing capability perspective: moderating role of marketing leadership team. Int. Market. Rev..

[9] Franco M., Minatogawa V., Durán O., Batocchio A., Quadros R. (2021). Opening the dynamic capability black box: an approach to business model innovation management in the digital era. IEEE Access.

[10] Guo F., Hartini H. (2023). Exploring the coupling relationship between dynamic marketing capability, strategic information management, and international startup performance. J. Inf. Syst. Eng. Manag..

[11] García-Ochoa C.P., De-Pablos-Heredero C. (2020). How business accelerators impact startup's performance: empirical insights from the dynamic capabilities approach. Intang. Cap..

[12] Shuja A., Yazdani N., Shuja A. (2021). Investigating resilience and performance of emergent financial technology startups endorsed by knowledge management. JISR Management and Social Sciences & Economics.

[13] Bitencourt C., de Oliveira Santini F., Ladeira W., Santos A.C.M.Z., Teixeira E. (2020). The extended dynamic capabilities model: a meta-analysis. Eur. Manag. J..

[14] Teece D.J. (2007). Explicating dynamic capabilities: the nature and microfoundations of (sustainable) enterprise performance. Strat. Manag. J..

[15] Eisenhardt K.M., Martin J.A. (2000). Dynamic capabilities: what are they?. Strat. Manag. J..

[16] Teixeira E.G., de Moura G.L., Lopes L.F.D., Marconatto D., Fischmann A. (2021). The influence of dynamic capabilities on startup growth. RAUSP Manag. J..

[17] Scuotto A., Cicellin M., Consiglio S. (2023). Social bricolage and social business model in uncertain contexts: insights for the management of minor cultural heritage in Italy. Meas. Bus. Excell..

[18] Buccieri D., Javalgi R., Çavuşgil E. (2020). International new venture performance: role of international entrepreneurial culture, ambidextrous innovation, and dynamic marketing capabilities. Int. Bus. Rev..

[19] Proskurnina N., Chala A.A. (2023). The theoretical foundations of the enterprise strategic management system. Bus. Inf..

[20] Barney J. (1991). Firm resources and sustained competitive advantage. J. Manag..

[21] Nascimento L., Reichert F., Janissek-Muniz R., Zawislak P. (2020). Dynamic interactions among knowledge management, strategic foresight and emerging technologies. J. Knowl. Manag..

[22] Gupta B.B., Panigrahi P. (2023). Analysis of the role of global information management in advanced decision support systems (DSS) for sustainable development. J. Global Inf. Manag..

[23] Demir A.L. (2022). Strategic management and performance of commercial banks in Turkey. J. Strat. Manag..

[24] Kafel T., Ziębicki B. (2021). Dynamics of the evolution of the strategic management concept: from the planning school to the neostrategic approach. J. Entrep., Manag. Innov..

[25] Suarez J. (2022). The hallmarks of strategic management accounting: seeking to support decision making processes. J. Bus. Manag..

[26] Taqatqa A. (2023). The impact of globalization on strategic management in Lebanon. Int. J. Strat. Market. Pract..

[27] Belias D., Rossidis I. (2021). Corporate Leadership and its Role in Shaping Organizational Culture and Performance.

[28] Lobo F., Akdere M. (2023). Proceedings of the International Conference on Industrial Engineering and Operations Management.

[29] Zadoia V., Charkina T. (2023). Strategic management in tourism: key aspects. Rev. Transport Econ. Manag..

[30] Lis T., Bajdor P., Ptak A. (2020).

[31] Civelek M., Krajčík V., Ključnikov A. (2023). The impacts of dynamic capabilities on SMEs' digital transformation process: the resource-based view perspective. Oeconomia Copernic..

[32] Kuděj M., Civelek M., Erben M., Masárová J., Kubálek J. (2023). Navigating global markets: the role of enterprise risk management and human resource management in SME international expansions. Equilib. Q. J. Econ. Econ. Pol..

[33] Diaz Tautiva J.A., Salvaj Carrera E., Vásquez-Lavín F., Ponce Oliva R.D. (2023). Understanding the role of institutions and economic context on entrepreneurial value creation choice. Oeconomia Copernic..

[34] Greenhalgh T., Robert G., Macfarlane F., Bate P., Kyriakidou O., Peacock R. (2005). Storylines of research in diffusion of innovation: a meta-narrative approach to systematic review. Soc. Sci. Med..

[35] Ledesma-Chaves P., Arenas-Gaitán J., Garcia-Cruz R. (2020). International expansion: mediation of dynamic capabilities. Market. Intell. Plann..

[36] Puthusserry P., Khan Z., Knight G., Miller K. (2020). How do rapidly internationalizing SMEs learn? Exploring the link between network relationships, learning approaches and post-entry growth of rapidly internationalizing SMEs from emerging markets. Manag. Int. Rev..

[37] Martin S.L., Javalgi R.R.G., Ciravegna L. (2020). Marketing capabilities and international new venture performance: the mediation role of marketing communication and the moderation effect of technological turbulence. J. Bus. Res..

[38] Ferreira J., Coelho A. (2020). Dynamic capabilities, innovation and branding capabilities and their impact on competitive advantage and SME's performance in Portugal: the moderating effects of entrepreneurial orientation. Int. J. Innovat. Sci..

[39] Wang F. (2020). Digital marketing capabilities in international firms: a relational perspective. Int. Market. Rev..

[40] Liu C.L., Zhang-Zhang Y., Ghauri P.N. (2020). The influence of internet marketing capabilities on international market performance. Int. Market. Rev..

[41] Deng P., Liu Y., Gallagher V.C., Wu X. (2020). International strategies of emerging market multinationals: a dynamic capabilities perspective. J. Manag. Organ..

[42] Reimann C., Carvalho F., Duarte M. (2021). The influence of dynamic and adaptive marketing capabilities on the performance of Portuguese SMEs in the B2B international market. Sustainability.

[43] Wójcik P., Ciszewska-Mlinarič M. (2021). The impact of cognitive and behavioral factors on the export performance: a dynamic capabilities perspective. Eur. Bus. Rev..

[44] Scuotto V., Nespoli C., Palladino R., Safraou I. (2022). Building dynamic capabilities for international marketing knowledge management. Int. Market. Rev..

[45] Buccieri D., Javalgi R., Jancenelle V.E. (2021). Dynamic capabilities and performance of emerging market international new ventures: does international entrepreneurial culture matter?. Int. Small Bus. J. Res. Entrep..

[46] Kim K., Lim G. (2022). International dynamic marketing capabilities of emerging-market small business on e-commerce. J. Theor. Appl. Electron. Commer. Res..

[47] Hu Y.P., Lee C.M. (2022). Impact of intellectual capital on international trade: knowledge management and business processes as intermediaries. Int. J. Innov. Res. Sci. Stud..

[48] Ciszewska-Mlinarič M., Siemieniako D., Wójcik P. (2024). International dynamic marketing capabilities and international performance during the pandemic: a study of export manufacturers from Poland. Int. Market. Rev..

[49] Rivero-Gutiérrez L., Cabanelas P., Díez-Martín F., Blanco-González A. (2024). How can companies boost legitimacy in international markets? A dynamic marketing capabilities approach. Int. Market. Rev..

[50] Weaven S., Quach S., Thaichon P., Frazer L., Billot K., Grace D. (2021). Surviving an economic downturn: dynamic capabilities of SMEs. J. Bus. Res..

